# The Epithelial Sodium Channel α subunit (α ENaC) alternatively spliced form "b" in Dahl rats: What's next?

**DOI:** 10.1186/1755-7682-3-14

**Published:** 2010-07-06

**Authors:** Marlene F Shehata

**Affiliations:** 1 Department of Cellular and Molecular Medicine, Faculty of Medicine, University of Ottawa, Ottawa, ON, Canada

## Abstract

**Background:**

The amiloride-sensitive Epithelial Sodium Channel (ENaC) is critical in maintaining Na^+ ^balance, extracellular fluid volume and long term blood pressure control. ENaC is composed of three main subunits α, β, & γ. While α ENaC is critical for channel functionality, β & γ ENaC maximize channel function. To date, there are four alternatively spliced forms of the α subunit of ENaC (α ENaC-a, -b, -c, & -d) that have been published in rats, in addition to the major α ENaC transcript. While α ENaC-a, -c & -d transcripts are low abundance transcripts compared to full-length α ENaC, α ENaC-b is a higher abundance and salt-sensitive transcript compared to full-length α ENaC.

**Presentation of the hypothesis:**

α ENaC-b protein, which is preferentially produced in Dahl R rats, to a greater extent on high salt diet, exerts a dominant negative effect on full-length α ENaC subunit by physically binding to and trapping full-length α ENaC subunit in the endoplasmic reticulum, and finally accelerating full-length α ENaC proteolytic degradation in a dose-dependent manner.

**Testing the hypothesis:**

1) To examine the mRNA and protein abundance of α ENaC-b relative to α ENaC full-length in kidney, lung, and taste tissues of Dahl rats. 2) To compare the expression (mRNA and protein) of α ENaC-b in kidneys of Dahl S and R rats on regular and high salt diet. 3) To examine the putative binding of α ENaC-b proteins to full-length α ENaC *in vitro *and to determine the impact of such binding on full-length α ENaC expression *in vitro*.

**Implications of the hypothesis:**

Our studies will be the first to demonstrate the over-expression of salt-sensitive α ENaC-b spliced form in kidney tissues of Dahl R rats at the expense of full-length α ENaC. The current proposal will provide highly novel insights into the putative mechanisms leading to ENaC hypoactivity in high-salt-fed Dahl R rats. Finally, findings from the present proposal will uncover a new mechanism by which alternative splicing may control the regulation of ENaC expression/function.

## Background

The amiloride-sensitive Epithelial Sodium Channel (ENaC) constitutes the major route for transporting the sodium ion (Na^+^) into the cell and hence, is critical in the maintenance of Na^+ ^balance, extracellular fluid volume and long term blood pressure control. ENaC is primarily composed of three subunits denoted by α, β, & γ. α ENaC subunit is critical for channel functionality, while β & γ subunits serve to maximize channel functionality. As such, being the most critical subunit in channel functionality, α ENaC is the focus of the present hypothesis. To date, there are four alternatively spliced forms of the α subunit of ENaC (α ENaC-a, -b, -c & -d) that have been published in rats, in addition to the major α ENaC transcript [1,2]. α ENaC-a, -c & -d transcripts are low abundance transcripts compared to full-length α ENaC and have been defined in terms of expression. Additionally, α ENaC-a has demonstrated non-functionality in oocytes as well as binding to ENaC blocker [1]. On the other hand, α ENaC-b is yet to be fully characterized, but appears to play a role in modulating ENaC, for the following reasons: a) α ENaC-b mRNA expression is significantly higher in Dahl salt-resistant (R) rats (with suppressed Na^+ ^transport related to ENaC [3,4]) *versus *Dahl salt-sensitive (S) rats [2] (with enhanced Na^+ ^transport related to ENaC [3,4]); b) α ENaC-b is a salt-sensitive transcript, because high *versus *normal salt diet caused a large increase in α ENaC b mRNA (P ‹ 0.05) levels in Dahl R rats [2]; c) unlike α ENaC-a, -c &-d, α ENaC-b mRNA levels are ~32 ± 3-fold higher than full-length α ENaC; and finally d) the splice site generating α ENaC-b is highly conserved across species. As such, our results demonstrate a clear shift from constitutive to alternative splicing of α ENaC pre-mRNA in favor of α ENaC-b formation, indicating an increased stability and/or preferential synthesis of the latter.

These facts, combined with the realization that large structural defects in an encoded protein (such as α ENaC-b) are usually associated with considerable effects on protein function that may extend to a drastic switch-off effect [5], in turn, emphasize the need for investment in basic research with a view to identifying novel targets for diagnosis, prevention and treatment of disorders related to ENaC dysfunction. The overall objective of these studies is to continue to examine the differential expression of α ENaC-b relative to full-length α ENaC in an array of tissues where ENaC plays a significant role. Subsequently, we will investigate whether α ENaC-b suppresses α ENaC expression/activity by binding to and/or accelerating proteolytic degradation of the latter in Dahl R rats (a model of suppressed ENaC). As such, we will pursue the following three specific aims:

### A. What is the comparative expression profile of α ENaC-b in kidney, lung and taste tissues of Dahl S and R rats fed regular salt diet?

We will continue to define the existence and later examine the mRNA and protein expression profiles of α ENaC-b compared to full-length α ENaC in Dahl rat kidney (already reported in our previous contribution [2]), lung and taste tissues where ENaC plays a significant role. Cell-specific expression of α ENaC-b might allow particular cell types to individually regulate ENaC expression/function.

### B. Is α ENaC-b over-expressed in response to salt loading in kidneys of Dahl R rats with suppressed ENaC activity compared to Dahl S rats with overly active ENaC?

We will narrow our studies to studying the impact of high dietary sodium on α ENaC-b expression in Dahl rat kidney tissues. We will continue our QRT-PCR experiments that demonstrated an enhanced mRNA [2] and will follow by Western analysis to examine protein expression of α ENaC-b in kidneys of Dahl R compared to S rats on high *versus *regular salt diet. We will then proceed to quantify α ENaC-b expression relative to full-length α ENaC in Dahl R *versus *S rats. These studies will show whether suppressed full-length α ENaC expression in Dahl R rats is consistent with an enhanced α ENaC-b mRNA and protein levels.

### C. Does α ENaC-b protein interact with and/or directly bind to full-length α ENaC and subsequently alter full-length α ENaC expression?

We will proceed to examine if α ENaC-b protein sequesters full-length α ENaC (by co-immunoprecipitation assays). The impact of α ENaC-b on full-length α ENaC expression has been elucidated (dose-dependent co-expression experiments) in cellular models (as recently reported in our studies [6]). These co-immunoprecipitation studies along with our previously reported western analyses will demonstrate the mechanism by which α ENaC-b contributes to a suppressed overall Na^+ ^transport related to ENaC in Dahl R rats on high Na^+ ^(e.g., by enhanced binding of α ENaC-b to full-length α ENaC, followed by proteolytic degradation of the latter).

## Presentation of the hypothesis

### General Hypothesis

α ENaC-b protein, which is preferentially produced in Dahl R rats, to a greater extent on high salt diet, exerts a dominant negative effect on full-length α ENaC subunit by physically binding to and trapping full-length α ENaC subunit in the endoplasmic reticulum, and finally accelerating full-length α ENaC proteolytic degradation in a dose-dependent manner.

### Specific Hypotheses

▪ α ENaC-b is differentially expressed in the kidneys, lungs and taste tissues of Dahl rats to individually regulate ENaC expression/function in these tissues.

▪ At the expense of full-length α ENaC expression levels, α ENaC-b expression (mRNA and protein) is enhanced in response to salt, in kidneys of Dahl R, but not S rats to suppress α ENaC expression/activity.

▪ α ENaC-b proteins trap full-length α ENaC in the endoplasmic reticulum and enhance full-length α ENaC proteolytic degradation in a dose-dependent manner and hence serve as dominant negatives on α ENaC expression/activity.

### Objectives

1. To examine the abundance of α ENaC-b relative to α ENaC full-length in kidney, lung, and taste tissues of Dahl rats.

2. To compare the expression (mRNA and protein) of α ENaC-b in kidneys of Dahl S and R rats on regular and high salt diet.

3. To examine the putative binding of α ENaC-b proteins to full-length α ENaC *in vitro *and to determine the impact of such binding on full-length α ENaC expression *in vitro*.

### Rationale

Owing to the established body of evidence pointing out the crucial role of alternatively spliced forms (particularly those lacking important functional domains) as dominant negative variants on full-length proteins [7-9], we chose to examine the putative role of alternatively spliced form α ENaC-b in ENaC regulation for the following **six **reasons: a) α ENaC-b mRNA levels are ~32 ± 3-fold higher than full-length α ENaC in kidneys of Dahl rats [2], indicating increased stability and/or synthesis of the former, b) α ENaC-b mRNA concentrations are significantly higher in Dahl R *versus *S rat kidneys on regular salt diet [2], c) At the expense of full-length α ENaC protein, Dahl R rats kidney tissues may demonstrate enhanced expression of α ENaC-b compared to the full-length α ENaC; d) α ENaC-b is a salt-sensitive transcript, because high *versus *normal salt diet caused a large increase in α ENaC-b mRNA levels (P < 0.05) in Dahl R rats [2,10], e) the splice site used to generate α ENaC-b is conserved in humans, f) α ENaC-b is translatable [2,6] (Translate tool^® ^http://ca.expasy.org/tools/dna.html). Consistent with the putative role of α ENaC-b in ENaC dysregulation, our previously reported results are consistent with a dominant negative effect imposed by α ENaC-b on full-length α ENaC [6]. Dahl R and S rats serve as attractive models to study the contribution of α ENaC-b on differential ENaC expression and activity. This is because on high salt diet, Dahl R, but not S rat kidneys have a suppressed Na^+ ^transport related to ENaC [3,4]. Our findings could provide novel targets for regulating α ENaC congener levels by manipulating the expression of the alternatively spliced form-b or by expressing an exogenous α ENaC-b.

Our current proposal examines three potential mechanisms by which α ENaC-b may regulate the renal full-length α ENaC by a dominant negative effect. The **first mechanism **is through suppressing α ENaC **transcription **via a shift from constitutive to alternative splicing of α ENaC pre-mRNA in favor of α ENaC-b formation. The **second mechanism **is through suppressing **translation **of full-length α ENaC more prominently in Dahl R ***versus ***S rats. The **third mechanism **is via an enhanced binding of α ENaC-b protein to the full-length α ENaC and accelerated degradation of the latter as a result of direct binding to α ENaC-b, a phenomenon that has been reported previously in several other channels and membrane proteins [7-9,11-13]. Enhanced binding of α ENaC-b to the full-length α ENaC might trap α ENaC in the endoplasmic reticulum and/or inhibit proper ENaC assembly and trafficking to the plasma membrane, resulting in the formation of non functional channels. On the other hand, suppressing α ENaC full-length protein expression will, in turn, disturb the proportions of the ENaC αβγ proteins to be translocated to the plasma membrane and ultimately hinder overall channel cell surface expression and activity.

Knowledge of the mechanism by which α ENaC-b regulates full-length α ENaC and possibly reduces expression/activity of ENaC in Dahl R rats and the subsequent genesis of salt-dependent hypertension [a disease that comprises a large subgroup (over 50%) of Canadian adults] would enhance the understanding of the basic regulation of ENaC and the pathophysiology of ENaC-associated disorders such as salt-sensitive hypertension. It may also create one or more specific targets for the development of novel anti-hypertensive drug or gene therapy.

### Testing the hypothesis

#### Animal models

Dahl S rats are used as a genetic model for enhanced ENaC activity. ENaC activity is twice in Dahl S kidney *versus *Dahl R kidney [3,4]. In contrast, Dahl R rats are the primary control strain with suppressed ENaC function. Usually, 8-10 rats per group are sufficient for statistical significance. Our research plan can be summarized as follows:

▪ We plan to examine the expression (mRNA and protein) of α ENaC-b relative to α ENaC wt in lung, kidney and taste tissues of Dahl S and R rats.

▪ We will subsequently proceed to investigate the effect of salt loading on the expression (mRNA and protein) of α ENaC-b in kidneys of Dahl rats. We already published the mRNA levels of α ENaC-b in kidney cortex of Dahl S *versus *R rats on normal and high salt diet [2]. We will continue by studying the mRNA levels and protein levels in kidney medulla.

▪ Finally, we will continue examining α ENaC-b heterodimerization with full-length α ENaC, and the impact of such heterodimerization on full-length α ENaC expression (using COS7 cells, antibodies, co-immunoprecipitation assays and dose-dependent expression studies [6]).

Experiments will be performed on male Dahl S and R rats (n = 24), 3-4 weeks of age, obtained from Harlan Sprague Dawley (Indianapolis, IN) and handled as previously described [2,6,14,15]. The rats will be divided into 4 groups (6 rats/group) by placing them on either regular (normal) (120 μmol Na^+^/g) or high-salt (8% NaCl or 1,370 μmol Na^+^/g, Teklad; Madison, WI) diet for four weeks. After 4 wks, blood pressure (BP) will be measured invasively by intra-arterial catheter and the average mean arterial pressure will be estimated. The animals will then be killed by decapitation and kidney, lung and tongue tissues will be removed and placed in cold methylbutane and then on dry ice. Tissues will be preserved at -80°C for later protein and RNA isolation. All experiments will be carried out in accordance with the guidelines of the University of Ottawa Animal Care Committee for the care and use of laboratory animals.

### Specific Methodology

#### Aim 1: What is the expression profile of α ENaC-b in kidney, lung and taste tissues of Dahl S and R rats?

To determine the mRNA and protein expression levels of α ENaC-b *versus *full-length α ENaC, we will isolate protein and total RNA from kidney, lung and taste tissues. Then we will proceed by performing western analyses and QRT-PCR to examine and later quantify α ENaC proteins and mRNAs. Detailed protocols are as follows:

##### Protein and RNA isolation

Whole kidney, lung and tongue tissues are homogenized with polytron. Total protein and total RNA are isolated using Trizol reagent (Invitrogen, CA, USA) according to the manufacturer's protocol. Isolated RNA is subjected to DNAse treatment for removing potential genomic DNA contamination using DNase treatment kit (Ambion). The availability of an antiserum specific for α ENaC proteins (directed against the N-terminus amino acid residues 46-68 of the rat α ENaC) will allow us to probe for these proteins in kidney, lung and tongue cell homogenates.

##### Reverse transcription

2 μg mRNA is reverse transcribed to first strand cDNA by superscript II RNase H- Reverse transcriptase (Invitrogen, CA, USA). To examine the co-existence of α ENaC wt and -b form, we will utilize wt primers that are common between α ENaC wt and -b form (no C-terminus sequence is shared among the two forms together). For QRT-PCR, we will use primers that are designed specifically to amplify the α ENaC -b form by including the nucleotide deletions (79 bp in α ENaC-b reverse primer). For α ENaC wt sequence, we will use the GeneBank accession number NM_031548 (α ENaC wt sequence was confirmed by our previously published study [15]), and for α ENaC -b, we will use the sequence previously reported by us [2].

##### Cloning

Full-length α ENaC and alternatively spliced form -b are cloned using DH5 α competent cells, and TOPO TA cloning kit^® ^(Invitrogen, ON, Canada). The resulting clones are sequenced using ABI prism 3100 (PE Applied Biosystems, Foster City, CA). Sequencing is performed using the DYEnamic ET Terminator kit according to the instructions provided by the manufacturer (PE Applied Biosystems, Foster City, CA). Sequencing products are purified (DyeEx 2.0 spin kit columns; Qiagen Canada, Mississauga, ON, Canada).

##### Quantitative real-time PCR

cDNAs are amplified using the previously described primers for wt α ENaC and α ENaC-b form. The relative amount of full-length α ENaC and alternatively spliced form mRNA are measured by quantitative real time RT-PCR using Roche light cycler and Fast Start DNA master SYBR Green I dye. Known plasmid concentration is used as a calibrator and water is used as negative control for each reaction. Experiments are done in triplicates for each form (full-length and -b form). Melting curve analysis is performed for each reaction to assess product specificity. Products are visualized on gel using gel electrophoresis assay to confirm the correct product sizes. Normalization is done using 3-phosphoglycerate-kinase (PGK) as a housekeeping gene.

##### Electrphoresis Analysis (Western Blot Analyses)

Concentrations for whole kidney, lung and tongue proteins (and proteins harvested from cultured cells under **aim 3**) are measured by Bradford. Proteins are then separated by SDS-PAGE resolving gels containing 10% acrylamide, and 4% of the same solution for stacking gels (NuPAGE Bis-Tris gels, Invitrogen). Briefly, 20 μg of proteins are mixed with 100 μl 2 × SDS-PAGE sample buffer containing 0.1% SDS (sodium dodecyl-sulphate (w:v)) and supplemented with β-mercaptoethanol. Before loading, SDS samples are heated at 100°C for 5 minutes. Samples are then transferred onto a nitrocellulose membrane (Invitrogen) and incubated with primary (directed against α ENaC N-terminus) at a 1:1000 dilution. Blots are washed thrice for 5 minutes each with PBST. For standard Western blotting detection, blots are incubated with either an anti-rabbit HRP-conjugated antibody (1:10,000, Amersham), for 1 h at 25°C. For improved Western blotting detection to avoid HC and LC signals, blots are incubated (1 h at 25°C) with a 1:5,000 dilution of Protein A-HRP (Amersham) or a 1:10,000 dilution of Protein G-HRP (Upstate Biotech.), prepared in blocking buffer. After washing three times at 25°C with PBST (5 min each), blots are developed with ECL Plus (Amersham). The marker used is high range Biorad ladder catalogue 161-0309.

Corresponding protein bands for α ENaC-b will be later sequenced for confirming the protein sequence of α ENaC-b. We will be using Cambridge Peptides^® ^for sequencing α ENaC proteins http://www.cambridgepeptides.com/index.html.

#### Aim 2: Is α ENaC -b over-expressed in Dahl R rats with suppressed ENaC activity compared to Dahl S rats with overly active ENaC in response to salt loading?

Protein, total RNA extraction, QRT-PCR and western blot analyses will be performed as described under Aim 1. Dahl rats fed either regular or high salt diet will be examined for α ENaC-b expression *versus *full-length α ENaC in kidney tissues.

### Statistical Analysis

Differences between the expression levels of full-length α ENaC and alternatively spliced form -b will be analyzed in Dahl S *versus *R rats on normal and high salt diet using two way ANOVA (SigmaSTAT^®^). The statistical test is two sided to identify strain and dietary differences, the data will be expressed as means and ranges, and differences of P values of less than 0.05 will be considered as significant.

#### Aim 3: Does α ENaC-b protein directly bind to full-length α ENaC and subsequently alter full-length α ENaC expression?

##### RNA extraction, RT-PCR and PCR

To amplify full-length α ENaC and α ENaC-b for expression studies, specific primers will be designed to flank the open reading frames of full-length α ENaC and -b forms. High fidelity Expand Long Range, dNTPack (Roche Applied Science^®^, Quebec, Canada) will be employed to amplify full-length α ENaC wt and -b spliced form. Touchdown PCR method will be employed to improve the efficiency of gene amplification as follows (start Tm at 68°C and the annealing temp reduced 2°C per cycle for the next cycles up to Tm = 58, then the remaining cycles at a 58°C annealing temperature). After amplification, the specific fragments of full-length α ENaC and α ENaC-b will be visualized in 1% agarose gels.

##### Plasmids and Generation of Constructs

PCR products for α ENaC wt and -b form will be ligated with with pCR^®^2.1-TOPO^® ^vector using the TOPO TA^® ^cloning kit (Invitrogen, CA, USA), with blue/white screening of DH5α competent cells on ampicillin selective plates. Positive clones will be cultured in LB broth. DNA extracted from these cultures using Qiagen DNA miniprep kit, will be sequenced using the M13 universal forward and reverse primers, in addition to embedded primers within α ENaC wt and -b forms to verify their full-lengths. Sequencing will be performed using the DYEnamic ET Terminator kit according to the instructions provided by the manufacturer (PE Applied Biosystems, Foster City, CA). The sequence of the cloned α ENaC-b form is compared to the one previously reported [1,2], and the sequence of the full-length α ENaC is compared to the one previously reported for Dahl rats [15].

Ligated pCR^®^2.1-TOPO^® ^- α ENaC wt clone will be digested using EcoR1, while that of α ENaC-b with EcoRV and HindIII and then subcloned overnight at 16°C into PCMV-sport6 vector (Invitrogen, CA, USA) downstream the T7 promotor using the EcoRI site for α ENaC wt and the EcoRV and HindIII sites for α ENaC-b.

##### Cell culture and transfection

COS-African green monkey kidney cells will be maintained in culture at 37°C/5% CO2 using Dulbecco's Modified Eagle's Medium (DMEM, Hyclone^® ^Thermo Fisher Scientific Laboratories, Logan, UT) supplemented with glutamine and 10% heat-inactivated foetal bovine serum (FBS, PAA Laboratories). Transfections will be performed using lipofectamine 2000 reagent (Invitrogen, Carlsbad, CA) and up to 50 μg DNA per 10 cm plate. Immediately before transfection the culture medium will be replaced with 5 ml of DMEM supplemented with 10% (v/v) fetal bovine serum. PCMV-sport6/α ENaC wt and b forms will be transfected into COS-7 cells separately at a concentration of 24 μg DNA/plate, as well as co-transfected together in a 1:1 ratio of α ENaC wt: α ENaC-b. For dose-dependent expression experiments, α ENaC and α ENaC-b form will be transfected into COS-7 cells separately at a concentration of 24 μg DNA/plate, as well as co-transfected together in a dose-dependant manner as follows (full-length α ENaC: α ENaC-b is (8: 1 μg, 8:3 μg, 8:6 μg, 8: 12 μg, 8: 18 μg, 8: 24 μg, 8: 30 μg, 8: 42 μg). The empty PCMV-sport6 vector at a concentration of 24 μg DNA/plate will be transfected as a control. Then the cells will be harvested for > 2 h at 4°C in RIPA lysis buffer (10 mM NaPO_4_, 150 mM NaCl, 1% deoxycholate, 1% Triton X-100, and 0.1% SDS, pH 7.2) supplemented with the protease inhibitor cocktail (Sigma, St Louis, MO). Proteins will be handled and separated by electrophoresis as described under Aim 1.

##### Co-immunoprecipitation Assay

Co-immunoprecipitation (Co-IP) will be employed to identify interaction between α ENaC-b and full-length α ENaC. Co-IP is conducted in essentially the same manner as a western blot (see aims 1 and 2 above). However, in a co-IP, α ENaC target antigen precipitated by the antibody against α ENaC "co-precipitates" a binding α ENaC-b complex from a lysate, i.e., the interacting protein is bound to the target antigen, which becomes bound by the antibody that becomes captured on a Protein A or G gel support (Pierce kit^®^).

##### Implications of the hypothesis

Depending on the results obtained from the proposed studies, we will proceed to examine the sub-cellular localization of α ENaC-b (if localized in the cytosolic and/or particulate fractions) using immunohistochemistry and the same antibodies used in the present proposal. Then, we will again employ immunohistochemistry to investigate if the co-localization of α ENaC-b with α ENaC wt is perinuclear (such as in endoplasmic reticulum) or closer to the plasma membrane. Results from these studies will demonstrate if heteromerization of α ENaC-b with α ENaC wt traps the latter in the endoplasmic reticulum, or it still allows α ENaC wt migration to the plasma membrane. Then, it would be interesting to examine the impact of α ENaC-b on ENaC cell surface expression. We will use the same procedure employed by Firsov and colleagues in [16], where α ENaC-b together with, β, and γ rat ENaC subunits will be tagged with the FLAG reporter octapeptide (DYKDDDDK) that is recognized by the anti-FLAG M_2 _(M_2_Ab) mouse monoclonal antibody (Kodak). Then, expression in Xenopus oocytes and binding assays (immunoprecipitation) will follow. Results from this set of experiments will reveal if α ENaC-b suppresses ENaC cell surface expression.

To this end, we will proceed and assess the overexpression of α ENaC-b in Dahl S rats where α ENaC-b expression is suppressed compared to Dahl R rats. A replication-defective adenovirus (AV) will be used as the vector for delivery of over-expression sequences, as previously described [17]. Injecting AV in the CNS maintains the high levels of AV for at least 28 days post i.c.v. because of a diminished immune response in the CNS *versus *the periphery. There are at least two areas of potential clinical importance for these studies. First, identification of spliced forms that impair the hyperactivity of ENaC to sodium in the kidney of Dahl S rats would identify specific targets for the development of novel antihypertensive drug therapy for salt-dependent hypertension. Second, as gene therapy delivery systems continue to evolve, we will be examining in vivo delivery of adenoviruses carrying α ENaC-b and then assessing the resultant blood pressure effects. This may eventually gain relevance as a possible "proof-of-concept" gene therapy.

## Conclusion

Our studies will be the first to demonstrate the over-expression of salt-sensitive α ENaC-b spliced form in kidney tissues of Dahl R rats at the expense of full-length α ENaC. The current proposal will provide highly novel insights into the putative mechanisms leading to ENaC hypoactivity in high-salt-fed Dahl R rats. Finally, findings from the present proposal will uncover a new mechanism by which alternative splicing may control the regulation of ENaC expression/function.

## Competing interests

The author declares no competing interests.

## Authors' contributions

The author conceptualized the hypothesis, wrote and reviewed the manuscript.
